# Retinal Blood Velocity and Flow in Early Diabetes and Diabetic Retinopathy Using Adaptive Optics Scanning Laser Ophthalmoscopy

**DOI:** 10.3390/jcm8081165

**Published:** 2019-08-03

**Authors:** Cherilyn Mae A. Palochak, Hee Eun Lee, Jessica Song, Andrew Geng, Robert A. Linsenmeier, Stephen A. Burns, Amani A. Fawzi

**Affiliations:** 1 Department of Ophthalmology, Feinberg School of Medicine, Northwestern University, Chicago, IL 60611, USA; 2 Chicago Medical School, Rosalind Franklin University of Medicine and Science, North Chicago, IL 60064, USA; 3 Department of Biomedical Engineering, Northwestern University, Evanston, IL 60208, USA; 4 School of Optometry, Indiana University, Bloomington, IN 47405, USA

**Keywords:** adaptive optics scanning laser ophthalmoscopy, optical coherence tomography angiography, diabetic retinopathy, diabetes mellitus, retina, blood flow

## Abstract

Using adaptive optics scanning laser ophthalmoscopy (AOSLO), we measured retinal blood velocity and flow in healthy control eyes and eyes of diabetic patients with or without retinopathy. This cross-sectional study included 39 eyes of 30 patients with diabetes (DM) with mild non-proliferative diabetic retinopathy (NPDR) or without retinopathy (DM no DR) and 21 eyes of 17 healthy age-matched controls. Participants were imaged with a commercial optical coherence tomography angiography (OCTA) device (RTVue-XR Avanti) and AOSLO device (Apaeros Retinal Imaging System, Boston Micromachines). We analyzed AOSLO-based retinal blood velocity and flow, and OCTA-based vessel density of the superficial (SCP), deep retinal capillary plexus (DCP), and full retina. Retinal blood velocity was significantly higher in eyes with DM no DR and lower in NPDR across all vessel diameters compared to controls. Retinal blood flow was significantly higher in DM no DR and lower in NPDR in vessel diameters up to 60 μm compared to controls. When comparing flow outliers (low-flow DM no DR eyes and high-flow NPDR eyes), we found they had a significantly different retinal vessel density compared to the remaining eyes in the respective groups. Retinal blood velocity and flow is increased in eyes with DM no DR, while these parameters are decreased in eyes with mild NPDR compared to healthy age-matched controls. The similarity of OCTA vessel density among outliers in the two diabetic groups suggests an initial increase followed by progressive decline in blood flow and OCTA vessel density with progression to clinical retinopathy, which warrants further investigation.

## 1. Introduction

Diabetic retinopathy (DR) is a microvascular complication affecting 35% of patients with diabetes and is the leading cause of preventable blindness in working age adults worldwide [1,2,3]. DR is associated with hemorheological and vascular changes that lead to impaired autoregulation early on in the disease followed by progressive attenuation of the retinal microcirculation, with the clinical outcome being retinal ischemia and angiogenesis [4]. Given that retinal vascular dysfunction is central to its pathophysiology, a large body of research has focused on retinal blood flow in DR [5,6,7]. These studies, however, have generated a great deal of controversy, particularly regarding the direction of change of retinal blood flow in the early stages prior to the onset of clinical manifestations of DR [8,9]. 

Studies using fluorescein angiography with videography (VFA) relied on the mean transit time to estimate retinal blood flow. While some of these studies found increasing blood flow with worsening DR [5,9,10], others reported initial decreased flow in diabetic eyes without DR followed by increased flow with the onset of non-proliferative diabetic retinopathy (NPDR) [11,12]. Laser Doppler techniques measure single vessel velocity and diameter to estimate retinal blood flow. Most Doppler studies reported increased diameters and decreased velocity in diabetic subjects with and without DR, yet their blood flow reports were conflicting [13,14,15,16,17,18,19]. Some reported increased flow in DM with no DR or NPDR compared to controls [13,14,15,20], others reported increased flow in NPDR but no change in DM with no DR [18], and others no significant flow change in either [16]. Color Doppler imaging (CDI) studies based on ultrasound measured the larger vessels at the optic nerve and similarly reported mixed results for the impact of diabetes on blood flow velocity [21,22,23,24]. Limitations of these techniques must be considered to attempt to resolve these conflicting findings. VFA, CDI, and Laser Doppler deduce total retinal flow indirectly from single vessel measurements. These techniques generally focus on vessels larger than 50 μm, and none of them are able to measure capillary flow. Modern techniques to image retinal vasculature include Doppler optical coherence tomography (OCT) and adaptive optics (AO) ophthalmoscopy. Doppler OCT offers non-invasive, in vivo assessment of all the major peripapillary retinal venous diameters and their blood velocity and thus measures the absolute total retinal blood flow from an eye [25,26]. Using this technology, researchers showed that retinal blood flow is significantly decreased in eyes with severe NPDR and PDR compared to controls [27,28,29]. Notably however, Doppler OCT focuses on measuring all large peripapillary vessels and does not specifically capture small caliber vessels in the parafoveal region. 

In contrast to previous imaging modalities which typically examined larger vessels, AO scanning laser ophthalmoscopy (AOSLO) allows imaging of blood velocity and flow across a wider range of vessel diameters [30,31,32]. Techniques to study the retinal microvasculature with AOSLO include high frame rate non-confocal imaging [33,34], single scan lines of a vessel (XT imaging) [35,36], and dual-channel scanning [37]. High frame rate non-confocal imaging and dual-channel scanning can measure flow velocity in capillaries <15 μm. The XT imaging AOSLO technique introduced by Zhong et al. measures velocity and flow in medium to large sized vessels (15–100 μm) [35]. 

In this study, we used AOSLO XT imaging to study the hemodynamic changes in medium (15–60 μm) to large-sized retinal vessels (60–100 μm), in patients who have DM without DR and those with mild NPDR, to resolve the controversy regarding retinal blood flow changes in the early stages of diabetes. We compared the vessel diameter, blood velocity, and volumetric flow in the retinal vasculature in diabetic subjects to those in age-matched controls and studied the parafoveal capillary density in these subjects using OCT angiography. 

## 2. Methods

### 2.1. Study Design and Human Subjects 

Patients were recruited in the Department of Ophthalmology at Northwestern University in Chicago, Illinois between July 2018 and March 2019. The study was approved by the Institutional Review Board of Northwestern University, followed the tenets of the Declaration of Helsinki, and was in accordance with the Health Insurance Portability and Accountability Act regulations. Written informed consent was obtained from all patients before image acquisition.

Inclusion criteria were healthy subjects, and subjects with diabetes and eyes with DM without DR or mild NPDR based on clinical assessment by board certified ophthalmologists. Inclusion criteria for healthy eyes included no history of ophthalmic disease confirmed by clinical examination. Eyes of subjects with diabetes with or without mild NPDR were included based on clinical exam and patient history, including no evidence of prior ocular therapeutic intervention for diabetes (e.g., surgery, intravitreal injection, or pan-retinal photocoagulation). Classification of eyes with mild NPDR were graded based on the range of the Early Treatment for Diabetic Retinopathy Study (ETDRS) scale [38,39]. Type 1 and type 2 diabetics were included. 

Eyes were excluded from the study if they had evidence of other ocular disorders (e.g., cataract, glaucoma, age-related macular degeneration). We excluded eyes with cataract graded above nuclear opalescence grade three or nuclear color grade three that may compromise image quality. We also excluded poor quality AOSLO images and optical coherence tomography angiography (OCTA) images with quality scores ≤ 5 and signal strength index (SSI) < 60. 

Electronic medical records were reviewed to extract demographic and clinical information. All patients underwent a dilated eye exam and axial length measurements through optical biometry at the same visit. All measurements were conducted by a single certified examiner using an IOLMaster 700 (ZEISS, Jena, Germany).

### 2.2. Optical Coherence Tomography Angiography (OCTA)

OCTA images of 3 × 3 mm^2^ centered on the fovea were acquired using the RTVue-XR Avanti system (Optovue Inc., Fremont, CA, USA) with split-spectrum amplitude-decorrelation angiography (SSADA) software [40]. SSADA detects flow by quantifying decorrelation of OCT reflectance between two consecutive B-scans at the same location on the retina. The specifications of the machine include an A-scan rate of 70,000 scans per second, a light source centered on 840 nm, and a bandwidth of 45 nm. *En face* OCT angiograms were used as a map to guide AOSLO imaging and to identify vessels of interest within the superficial retinal vasculature. We used the periarterial capillary-free zone to distinguish arteries from veins [41]. Parameters regarding vessels of interest are further explained under the AOSLO XT imaging methods section. 

We then used the built-in AngioVue Analytics software (version 2017.1.0.151) to quantify the “parafoveal” vessel density of the superficial capillary plexus (SCP), deep capillary plexus (DCP), and full retina. The “parafovea” was defined as an annulus centered on the fovea with inner and outer ring diameters of 1 and 3 mm, respectively. Vessel density was defined as the area occupied by vessels and microvasculature and is reported as a percentage of the total area. 

### 2.3. Adaptive Optics Scanning Laser Ophthalmoscopy (AOSLO)

AOSLO imaging was done using the Apaeros Retinal Imaging System (Boston Micromachines Corporation, Cambridge, Massachusetts, USA) [42]. It uses a 97 actuator ALPAO DM (ALPAO SAS, Montbonnot, France) with 25 µm of stroke for wavefront correction, and two superluminescent diodes (SLD) as light sources centered at 790 and 850 nm, with respective bandwidths of 15 and 20 nm. Imaging was performed using the 790 nm source and wavefront sensing was performed using the 850 nm source, with a combined power at the eye of approximately 130 µW. Split-detection images were also acquired using two separate non-confocal detectors. Image intensity was determined by the difference of the non-confocal detector signals divided by their sum.

We acquired *en face* images of 1.5° × 1.77° dimension focused on vessels. We focused the machine at the center of the lumen in vessels of interest and acquired multiple 2 s videos of 60 frames (30 frames/s) to ensure inclusion of flow variations throughout the cardiac cycle.

### 2.4. AOSLO XT Imaging

**Figure 1:**
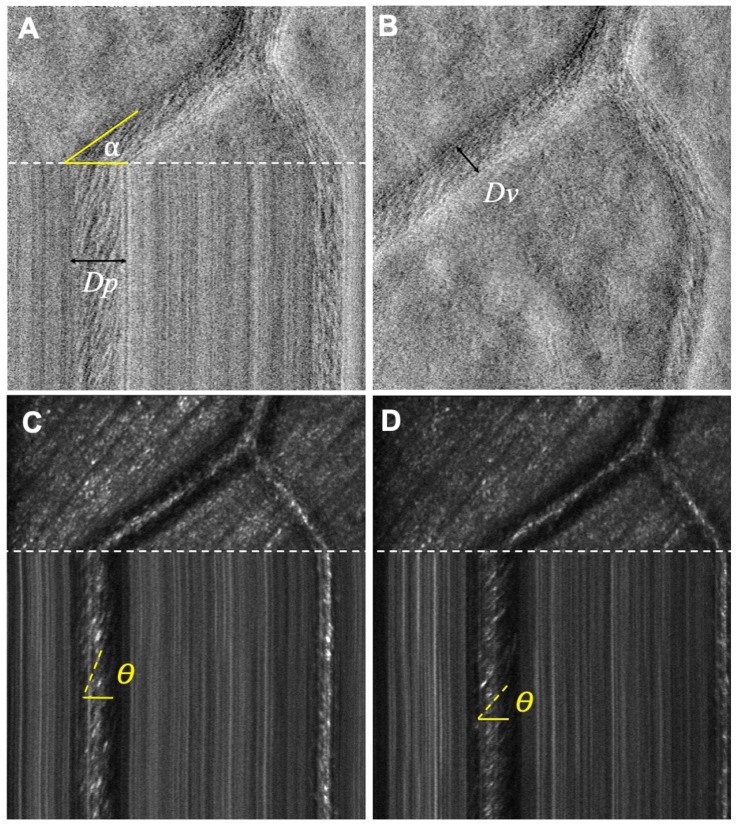
Adaptive Optics Scanning Laser Ophthalmoscopy (AOSLO) split-detector and confocal images. (**A**) Single-frame split-detector images showing XT image with the scan intersecting the blood vessel at angle alpha. The vessel lumen width was determined from the XT scan (*Dp*). (**B**) XY image showing the direct diameter of the vessel (*Dv*). (**C**,**D**) AOSLO confocal images showing the theta angle of RBC slopes at different phases of the cardiac cycle. Shallower slopes indicate higher velocity.

To measure blood velocity and flow, we employed XT imaging as described by Zhong et al. [35,36]. Vessels of interest, either arteries or veins, were identified from the relevant 3 × 3 mm^2^ OCTA image centered on the fovea. The ultimate goal in XT imaging was to obtain pictures with well-delineated vessel walls and clear streaks focused on the center of the vessel lumen. Briefly, the default mode of scanning in the AOSLO uses a horizontal scanner that moves down vertically during image acquisition. As the scanner moves down, lines of information are taken in succession and stitched together to form a full 2-dimensional image in the frontal plane of the retina—this type of image is referred to as an XY image. In an XT image, the vertical scanner is halted momentarily at a point of interest and takes lines of images sequentially at the same location. While XY images have two spatial dimensions, the two axes of the frontal plane, XT images have one spatial dimension (horizontal) and one temporal dimension (acquisition sequence). In practice we obtain video frames which are split between XY images (Figure 1B) and XT (Figure 1A,C,D). This dual mode video frame simplifies detection of eye movements by comparing the XY portion of sequential video frames. When properly focused on the vessel lumen, XT images will produce diagonal streaks (Figure 1) which represent erythrocyte flow. Using the slopes of the erythrocytes on the XT portion of our images, we can then extract the spatial and temporal information to calculate the velocity of these cells. While temporal information can be measured between successive XY images, the frame rate used (30 Hz) is too limited to track individual blood cells in the medium to large-sized vessels (15–105 µm diameter) vessels we targeted. This limitation is overcome by XT imaging, which uses a 15 kHz horizontal line rate. 

Several intrinsic qualities of the vessels ensured high accuracy measures of erythrocyte velocity and were set as criteria for vessels of interest. These are the (1) alpha angle, (2) distance from bifurcations, and (3) vessel size. The alpha angle influences accuracy because the scanner is fixed and does not have rotating capabilities. An alpha angle between 50°–90° causes two things to occur: The horizontal component of velocity (*Vp*) is too small along the X-axis, and the horizontal distance traveled by particles (*Dp*) decreases, thus decreasing the size of the streak. Both of these factors contribute to increased uncertainty in the horizontal component of blood velocity and were; therefore, avoided. Secondly, the smaller the vessel size, the more difficult it is to center on the lumen, in the presence of small eye movements, resulting in poor visualization and fewer streaks. Thus, we focused on medium sized vessels larger than 15 µm in diameter. Lastly, we avoided measuring too close to bifurcations to avoid uneven flow distributions across the vessel, which affects the velocity and flow [35].

### 2.5. XT Imaging Analysis: Blood Velocity and Flow Calculations

Flow and velocity measurements were completed by three independent graders (CAP, HEL, and JS). Two of the graders were masked to the health status of the eye to remove bias. Image quality was determined by the lack of ocular movement (stationary structures presented as straight vertical lines) and streak contrast and clarity. We then followed the protocol set forth by Zhong et al. [1,2], with a few adaptations. Briefly, to calculate the velocity of erythrocytes in the lumen, the slopes of the streaks in the confocal XT image were measured using angle θ (0 < θ < π/2) against the horizontal line (Figure 1). Based on this slope, the horizontal vector of velocity (*Vp*) is given by:

(1) $$Vp\;=\;\frac{f\;\cdot \;\cot\theta }{k}$$

where *f* is the horizontal frame rate (15 kHz) to calibrate for time, and *k* is the calculated magnification taking into account the axial length. The velocity within the axis of the vessel is then:

(2) $$Vax\;=\;\frac{Vp}{\cos\alpha }$$

To calculate flow, we measured the diameter of the lumen and alpha angle on the split-detector images, which differed from Zhong’s protocol. The use of split-detector images (versus confocal) allowed better visualization of the inner and outer vessel walls. Vessel lumen diameter (*Dv*) was calculated using the measured alpha angle and the horizontal distance of the XT image (*Dp*) based on Equation (3). The measurement was confirmed by directly measuring the vessel lumen diameter in the XY image (Figure 1). Given the lumen diameter and velocity, blood flow (*Q*) was calculated based on Equation (4).

(3) Dv = Dv · sinα

(4) $$Q\;=\;\frac{Vax\;\cdot \;\pi \;\cdot {\;Dv}^{2}}{4}$$

In selecting XT images, we sought to minimize factors that could artificially change flow and velocity such as ocular movement. In the XT portion of the images, static structures should appear as straight lines. Any deviations indicate small ocular movements and these images were discarded from the analysis. We used at least three particle streaks centered in the lumen with the shallowest slopes to measure the average maximum velocity. In order to account for the variations of velocity and flow due to pulsation, we selected images with either maximum or minimum RBC slopes from multiple image sequences taken at the same location and categorized them into two sets of images (minimum vs. maximum, 2 to 4 images each). We used the average of the minimum and maximum calculated velocities from these two sets of non-sequential frames in order to determine the average velocity for each vessel. All images were exported and measured on ImageJ (developed by Wayne Rasband, National Institutes of Health [NIH], Bethesda, MD, USA; available in the public domain at http://rsb.info.nih.gov/ij/index.html).

### 2.6. Statistical Analysis

Statistical analysis was performed using IBM SPSS software version 25 (IBM SPSS Statistics; IBM Corporation, Chicago, IL, USA) with the significance level set at 0.05. Descriptive statistics were calculated for all groups, and variables were expressed as mean ± standard deviation (SD). Characteristics of the study population were compared by independent *t*-test for continuous parameters and by chi-square test for categorical parameters. Pearson correlations were performed to determine the correlation between velocity and diameter of each group, and to determine whether the degree of correlation differed across the groups. One-way analysis of variance (ANOVA) was used to compare the difference between all groups. Populations were tested for equality of variance using the Levene test for homogeneity of variances. If the distributions failed, then Welch ANOVA was performed to correct for inhomogeneity of variances. Tukey or Games-Howell post-hoc tests were performed following one-way or Welch ANOVA, respectively. The main outcome measures were vessel diameter, blood velocity and flow.

## 3. Results

### 3.1. Subjects

The overall demographic, clinical, and disease-related characteristics are reported in Table 1. Of the 65 eyes that were imaged for this study, five were excluded due to motion artifact on XT images, leaving a total of 60 eyes from 47 participants (30 diabetic, 17 healthy controls). There were no significant differences in age or gender between groups. There were no significant differences in diabetes type, disease duration, and hemoglobin A1c (HbA1c) between subjects with diabetes with and without mild NPDR. Additional demographics and disease related characteristics are reported in Table 1.

### 3.2. OCTA Parafoveal Vessel Density

OCTA parafoveal densities of the SCP, DCP, and full retina were significantly different between the groups. The overall results are reported in Table 1.

### 3.3. Comparison of AOSLO Blood Velocity across Groups

**Figure 2:**
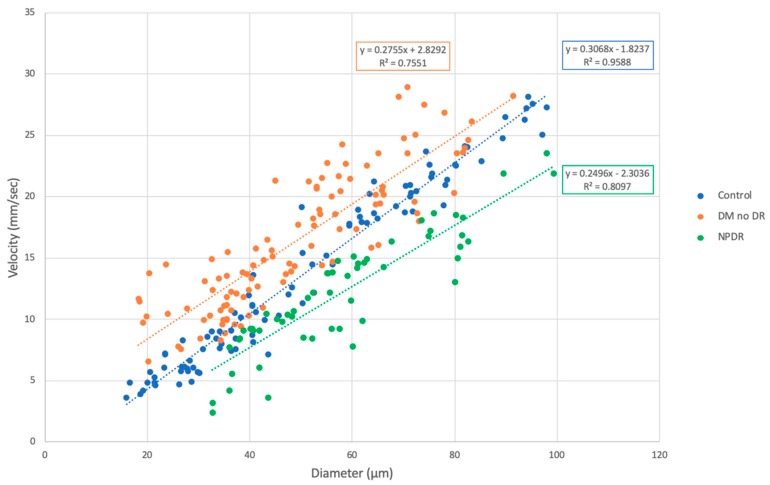
Blood velocity in retinal vessels (arterioles, venules, and capillaries) by vessel diameter, comparing healthy controls, diabetes without retinopathy, and non-proliferative diabetic retinopathy. A significant correlation was observed between velocity and vessel diameter in both arteries and veins within groups. Linear regression coefficients (*r*) were 0.979 for controls, 0.880 for DM without DR, and 0.900 for NPDR eyes. One-way ANOVA comparing regression coefficients showed a significant difference across the three groups (*p* = 0.007). Abbreviations: DM = diabetes mellitus, DR = diabetic retinopathy, NPDR = non-proliferative diabetic retinopathy.

As expected, we found a significant correlation between mean velocity and diameter in all groups, where velocity increased with increasing diameter (Figure 2). This trend was found to be true in arteries and veins. Linear regression coefficients (*r*) were 0.979 for controls, 0.880 for DM without DR, and 0.900 for NPDR eyes. One-way ANOVA comparing the regression coefficients showed a significant difference between the three groups (*p* = 0.007). One-way ANOVA showed a statistically significant difference in velocity across all vessel diameters comparing all three groups (Table 2). Eyes with DM without DR had significantly higher velocity than control vessels up to 60 μm and higher than NPDR for all diameters. NPDR eyes had velocities significantly lower than controls and DM no DR eyes across all vessel diameters (Table 2). When we examined these differences by arterioles and venules, we found that eyes with DM without DR had significantly higher velocity than controls arteries up to 60 μm and veins up to 30 μm (Table 3 and Table 4). Eyes with DM without DR had significantly higher velocity than NPDR across all arterial and venous diameters. NPDR eyes had velocities significantly lower than controls especially in arteries > 60 μm and veins > 50 μm.

### 3.4. Comparison of Blood Flow Across Groups

**Figure 3:**
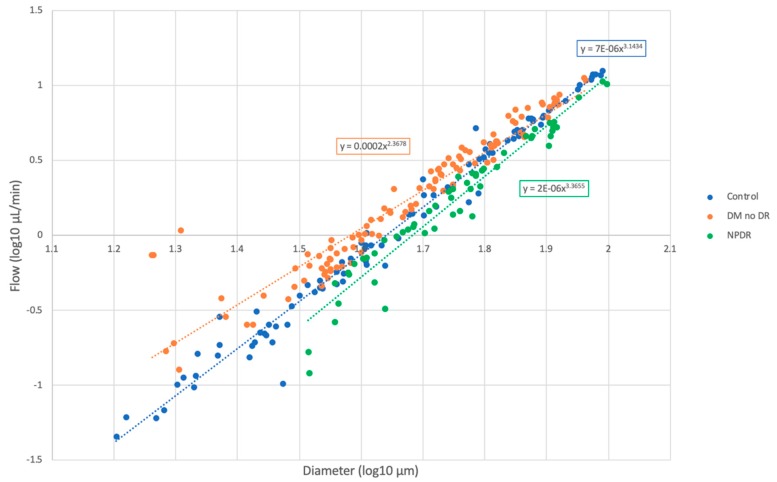
Log–log scale of blood flow in arterioles and venules by vessel diameter, comparing healthy controls, diabetes without retinopathy, and non-proliferative diabetic retinopathy. The exponent for blood flow as a function of diameter varied about a cubic relation depending on the patient group. Linear regression coefficients (*r*) were 0.991 for controls, 0.947 for DM without DR, and 0.969 for NPDR eyes. Abbreviations: DM = diabetes mellitus, DR = diabetic retinopathy, NPDR = non-proliferative diabetic retinopathy.

Blood flow increased with vessel diameter for all subject groups, as shown on a log–log scale (Figure 3). Regression coefficients (*r*) for this relationship were 0.991 for controls, 0.947 for DM without DR, and 0.969 for NPDR eyes. One-way ANOVA showed a statistically significant difference in mean flow in smaller vessels (up to 60 μm), when comparing the three groups (Table 5). Eyes with DM without DR had significantly higher flow than controls and NPDR in vessels up to 60 μm. NPDR eyes had flow significantly lower than DM no DR in vessels up to 60 μm and lower than controls in vessels greater than 60 μm. Blood flow was significantly different in arteries up to 60 μm and veins up to 30 μm, when comparing the three groups (Table 6 and Table 7).

### 3.5. Relationship between Velocity and Flow on AOSLO and Capillary Density on OCTA 

**Figure 4:**
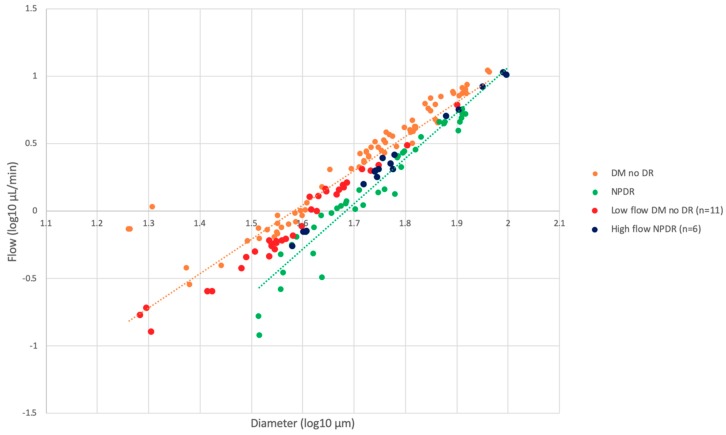
Log–log scale comparison of blood flow and diameter with OCT vessel density, comparing diabetes without retinopathy and non-proliferative diabetic retinopathy. Graph highlighting the distribution of the “high” and “low-flow” outliers as described in the text. Abbreviations: DM no DR = diabetes mellitus with no diabetic retinopathy, NPDR = non-proliferative diabetic retinopathy.

In order to better understand the variations in flow across groups, we examined the vessels that were outliers in flow for each group, defined as being more than one standard deviation (SD) away from the mean for a specific diameter in a disease group. We determined the relevant eyes and examined their OCTA vessel density. In the DM no DR group, 13 eyes (13 subjects) had the lowest flow measurements (0.13–6.11 μL/min), while eight eyes (eight subjects) had the highest flow measurements in the NPDR group (0.55–10.65μL/min). Two eyes from each group were excluded due to poor OCTA scan quality, leaving 11 “low-flow” DM no DR and six “high-flow” NPDR eyes for further analysis (Figure 4). Independent t-tests showed no significant difference between the “low-flow” DM with no DR and the “high-flow” NPDR groups when comparing their OCTA parafoveal densities and clinical characteristics (Table 8). The 11 “low-flow” DM no DR eyes had significantly lower mean full retina density than the remaining DM no DR group (53.81 vs 57.02; *p* = 0.021) (Figure 4). The six “high-flow” NPDR eyes had higher mean full retina density compared to the remaining NPDR group (55.28 vs 53.00), however this difference was not significant (*p* = 0.497). 

### 3.6. Flow Measurement Precision

To confirm the accuracy of the blood flow measurement, we quantified the total flow in vessels before and after a bifurcation in each of the three patient groups. The calculated time-averaged blood flow rate before and after the bifurcations were consistent with physical expectations (Table 9) [43].

## 4. Discussion

Using AOSLO XT imaging, we found that blood flow in eyes with DM without DR was significantly higher than controls, contrasting with significantly decreased flow in eyes with NPDR. For blood velocities, the trends were similar, showing statistical significance especially in smaller vessels. Our results distinguish velocity and flow changes in eyes with DM without DR from mild NPDR, which had not been possible in previous studies [13,15,16,18,20,23,44]. Using LDV, researchers found increased retinal blood flow and higher variance of flow in DM without DR compared to controls [15]. Wang et al. used Doppler OCT and found that eyes with DM no DR had total retinal blood flow within the normal range, although these eyes had significantly higher flow relative to PDR [26,45]. One potential explanation for this discrepancy between studies could be explained by our finding that small vessels (<60 μm) show the most significant differences in flow in eyes with DM no DR. Using AOSLO allowed us to reveal these differences as we were able to measure absolute blood flow across a wider range of vessel diameters (15–100 μm) than previously possible using other techniques. 

Our results suggest that during the early stages of diabetes, before the appearance of clinical retinopathy, there is an increase in retinal blood velocity and blood flow, as observed in the DM without DR group. This increase in flow, along with increased shear rate, could trigger cumulative endothelial damage in these eyes [46]. Over time, this damage may ultimately result in capillary closure, decreased retinal vessel density, and, ultimately, to decreased velocity and flow, as seen in eyes with mild NPDR in our study. To better understand the relationship between flow and vessel density during the transition from no DR to mild NPDR, we compared OCTA-derived measures of vessel density to the flow results. While blood flow was elevated in eyes with DM without DR and decreased in NPDR, we identified a subgroup of vessels that were intermediate between the two groups (Figure 4). We focused on eyes in the DM with no DR group that were 1 SD below the group means (“low-flow”) and NPDR eyes that were higher than the population means (“high-flow”). We found that the 11 “low-flow” DM no DR eyes had significantly lower full retina vessel density than the remaining DM no DR group, while the six “high-flow” NPDR eyes had higher full retina vessel density compared to the remaining NPDR group, though not significant. The non-significant difference in the high-flow NPDR group could be attributable to the relatively smaller sample size of the NPDR eyes. More interestingly, we found that these “low-flow” no DR eyes had similar diabetes duration and OCTA measurements to the “high-flow” NPDR, suggesting they may represent a transitional state and that these eyes are potentially at risk for imminent clinical progression (Table 8). The overall trend of this dataset suggests that changes in blood flow and vessel density reflect vascular remodeling and a gradual process of transition to clinically evident retinopathy. This is consistent with our previously reported finding of significantly increased retinal blood flow and decreased OCTA vessel density at the superficial capillary plexus (SCP) prior to the onset of clinical DR [47,48]. It is possible that the increased capillary flow is a compensatory mechanism in these eyes that enables them to meet the metabolic needs of the retina in the setting of decreased capillary density. The ability of the vasculature to respond and to compensate may explain why these eyes do not show clinical retinopathy. It is then possible that when this compensatory flow mechanism reaches its limit and is no longer able to compensate for the continued capillary loss, that the clinical manifestations of DR appear along with further decline in flow and velocity. It remains unclear whether one of these parameters (flow vs. vascular density) changes prior to the other and drives the vascular pathology. This question can only be answered by longitudinal, large scale, multimodal AOSLO and OCTA studies. 

The relationship between vessel diameter and flow is described by Murray’s law of branching vasculature, which predicts a cubic relationship [49]. In close agreement, we found that the mean blood flow varied with vessel diameter in a near cubic relationship in normal control eyes (for power fit, exponent = 3.15, Figure 3). The fitting exponent for DM without DR deviated from this relationship (exponent = 2.37) and was lower than controls. The fitting exponent for NPDR was different than DM without DR (exponent = 3.37) and was higher than controls. Deviations from Murray’s law in the retinal circulation have been seen previously and are suggested to be related to the Fåhræus-Lindqvist effect, which takes into account the change in blood viscosity by vessel diameter as opposed to the assumption of constant viscosity independent of diameter by Murray [50]. Our findings add further evidence to the notion that changes in blood viscosity and vessel properties, such as rigidity and diameter, may result in deviations from Murray’s law in pathologic situations [51]. Diabetes is known to increase blood viscosity and vessel rigidity due to intimal wall thickening [6], resulting in an increased fitting exponent, which we found in the NPDR group. The decrease in the fitting exponent seen in DM with no DR is interesting because it suggests that physiological factors other than viscosity and vessel rigidity could potentially play a role in this group of subjects.

The strengths of our study include AOSLO XT imaging, which uniquely allowed us to non-invasively measure absolute blood velocity and flow across a wider diameter range compared to previously used imaging techniques. Because of the video rate acquisition, we were able to capture and average changes occurring over the cardiac cycle. We also acknowledge several limitations of our study. One limitation is that we used the minimum and maximum velocities to determine an average velocity rather than analyzing sequential images to study the pulsatile dynamic changes in velocity. Image acquisition is intensive, requires trained personnel, and currently precludes the routine implementation of this approach in a clinical environment. In addition, the analysis is time consuming and may benefit from automated software tools. Another limitation is the relatively modest sample size of patients with mild NPDR, a limitation of our tertiary retina referral practice and our strict inclusion criteria of treatment-naïve eyes without macular edema. Due to the inflexible orientation of the AOSLO scanner, we were also limited to imaging vessels that were aligned with the direction of scanning. Another limitation is the sensitivity of the system to ocular saccades, which blur the XT image precluding measurements. Although current developments are underway to correct for blur related to eye motion, these have only recently been demonstrated in mice [52]. In some participants (13 out 47), two eyes were included in the analysis but in the reminder 34 cases, only one eye were considered. In nearly all subjects, both eyes were imaged. However, upon image analysis, we found that images were best from one eye, typically the first eye imaged. This may be due to decreased patient effort and focus over the prolonged time of imaging, resulting in poorer quality images of the second eye. We were unable to capture vessel diameters < 31 μm in NPDR eyes, and thereby unable to measure velocity and flow in the smallest capillaries. This was related to the greater number of blurred XT images in these smaller vessels, likely due to relatively unstable fixation in the NPDR group. Another explanation for this observation is that those with NPDR eyes may have had fewer available small capillaries that are aligned with the scanner, likely due to increased capillary closure. Finally, we did not measure or account for blood pressure or glucose level at the time of imaging, which may potentially contribute to measurement variability [11,53,54] and acknowledge that this should be recorded in future studies immediately prior to imaging.

## 5. Conclusions

In conclusion, using AOSLO we found that patients with DM without DR had significantly increased retinal vascular velocity and flow, while those with mild NPDR had significantly decreased measurements compared to controls. Future directions include long-term follow up of the subjects with DM without DR to study whether the decline in retinal blood velocity and flow could predict the onset of retinopathy in an individual subject and whether the rate of decreased flow can predict the rate of future progression of DR. Further studies to compare AOSLO-based capillary velocity and flow with OCTA-measured parameters such as parafoveal densities may provide additional insights into the nature of the interactions between retinal blood flow, capillary loss, and retinopathy progression. 

## Figures and Tables

**Table 1:** Patient demographics, clinical and diabetes characteristics, and capillary measurements on optical coherence tomography angiography.

	Healthy Controls	DM no DR	NPDR	*p*
Patients, *n*	17	21	9	
Eyes, *n*	21	26	13	
Vessel segments, *n*	94	110	56	
Vessel diameter range (μm)	15.98–97.93	18.27–91.88	32.71–99.45	
Age, mean ± SD,	57.7 ± 11.5	49.1 ± 16.3	47.6 ± 13.6	0.119
range	29–74	19–71	32–69	
Sex				0.069
Female, *n* (%)	13 (76%)	10 (48%)	3 (33%)	
Male, *n* (%)	4 (23%)	11 (52%)	6 (66%)	
DM type				0.398 *
Type 1, *n* (%)	n/a	6 (29%)	4 (44%)	
Type 2, *n* (%)	n/a	15 (71%)	5 (56%)	
Disease duration in years, mean ± SD	n/a	9.3 ± 7.6	15.2 ± 8.5	0.070 *
HbA1c, mean ± SD	n/a	7.3 ± 1.9	7.3 ± 1.1	0.998 *
Lens status				0.943
Clear	8 (47%)	11 (52%)	5 (56%)	
Cataract	8 (47%)	9 (43%)	3 (33%)	
Pseudophakic	1 (6%)	1 (5%)	1 (11%)	
Hypertension, *n* (%)	1 (6%)	9 (43%)	3 (33%)	–
Parafoveal vessel density (%)				
SCP, mean ± SD	47.97 ± 3.95	45.15 ± 4.49	43.23 ± 3.80	0.01
DCP, mean ± SD	53.57 ± 3.19	50.12 ± 3.50	47.59 ± 3.86	0
Full retina, mean ± SD	57.69 ± 3.35	55.55 ± 3.80	54.25 ± 2.67	0.023
OCTA SSI, mean ± SD	68.85 ± 6.15	67.45 ± 4.24	69.55 ± 6.62	0.522

Abbreviations: DM = diabetes mellitus, DR = diabetic retinopathy, NPDR = non-proliferative diabetic retinopathy, SCP = superficial capillary plexus, DCP = deep capillary plexus, SD = standard deviation, n/a = not applicable. *p*-value represents one-way ANOVA between all three groups. * *p*-value represents independent t-test or Chi-square test between DM without DR and NPDR. The healthy controls were not included.

**Table 2:** Summary of blood velocity in arterioles and venules by vessel diameter groups, comparing healthy controls, diabetes without retinopathy, and non-proliferative diabetic retinopathy.

	Control mm/s (*n* = 94)	DM no DR mm/s (*n* = 110)	NPDR mm/s (*n* = 56)	ANOVA *p*	Control vs DM no DR *p*	Control vs NPDR *p*	DM no DR vs NPDR *p*
<30 μm (*n* = 34)	3.60–8.32, 5.59 (23)	6.54–14.49, 10.42 (11)	n/a	0.000			
31–40 μm (*n* = 62)	5.64–13.58, 9.08 (20)	8.29–15.47, 11.51 (31)	2.42–9.23, 6.68 (11)	0.000	0.000	0.020	0.000
41–60 μm (*n* = 75)	7.13–19.18, 13.35 (15)	10.95–24.28, 17.67 (36)	3.59–15.13, 10.66 (24)	0.000	0.000	0.032	0.000
>61 μm (*n* = 89)	17.84–28.15, 21.98 (36)	15.88–28.94, 22.88 (32)	9.85–23.54, 16.70 (21)	0.000	0.499	0.000	0.000

Blood velocity, expressed as range in mm/s, mean (*n*), comparing different vessel diameters across groups. *n* equals number of measured vessel segments. *p* values were obtained using ANOVA and post-hoc tests. Abbreviations: DM = diabetes mellitus, DR = diabetic retinopathy, NPDR = non-proliferative diabetic retinopathy, n/a = not applicable.

**Table 3:** Summary of arteriolar blood velocity by vessel diameter groups, comparing healthy Controls, diabetes without retinopathy, and non-proliferative diabetic retinopathy.

	Control mm/s (*n* = 55)	DM no DR mm/s (*n* = 79)	NPDR mm/s (*n* = 47)	ANOVA *p*	Control vs DM no DR *p*	Control vs NPDR *p*	DM no DR vs NPDR *p*
<30 μm (*n* = 13)	4.22–7.15, 5.51 (9)	6.54–14.49, 10.59 (4)	n/a	0.001			
31–40μm (*n* = 34)	7.98–11.14, 9.42 (9)	9.41–15.47, 12.07 (18)	2.42–9.23, 7.17 (7)	0.000	0.000	0.169	0.006
41–60μm (*n* = 57)	10.33–19.18, 14.10 (9)	12.69–24.28, 18.02 (28)	6.06–15.13, 11.34 (20)	0.000	0.025	0.113	0.000
>60μm (*n* = 77)	17.84–28.15, 22.43 (28)	16.06–28.94, 22.98 (29)	9.85–23.54, 16.82 (20)	0.000	0.801	0.000	0.000

Blood velocity, expressed as range in mm/s, mean (*n*), comparing different arteriolar diameters across groups. *n* equals number of measured vessel segments. *p* values were obtained using ANOVA and post-hoc tests. Abbreviations: DM = diabetes mellitus, DR = diabetic retinopathy, NPDR = non-proliferative diabetic retinopathy, n/a = not applicable.

**Table 4:** Summary of venular blood velocity by vessel diameter groups, comparing healthy controls, diabetes without retinopathy, and non-proliferative diabetic retinopathy.

	Control mm/s (*n* = 39)	DM no DR mm/s (*n* = 31)	NPDR mm/s (*n* = 9)	ANOVA *p*	Control vs DM no DR *p*	Control vs NPDR *p*	DM no DR vs NPDR *p*
<30 μm (*n* = 24)	3.60–8.32, 5.75 (16)	7.56–13.79, 10.09 (8)	n/a	0.000			
31–50 μm (*n* = 32)	7.13–13.58, 9.40 (12)	8.29–17.75, 11.51 (15)	3.21–9.05, 5.98 (5)	0.000	0.066	0.026	0.000
> 50 μm (*n* = 23)	14.47–25.05, 18.84 (11)	14.43–26.86, 19.45 (8)	7.81–14.29, 9.95 (4)	0.001	0.930	0.001	0.001

Blood velocity, expressed as range in mm/s, mean (*n*), comparing different venular diameters across groups. *n* equals number of measured vessel segments. *p* values were obtained using ANOVA and post-hoc tests. Abbreviations: DM = diabetes mellitus, DR = diabetic retinopathy, NPDR = non-proliferative diabetic retinopathy, n/a = not applicable.

**Table 5:** Summary of blood flow in arterioles and venules by vessel diameter groups, comparing healthy controls, diabetes without retinopathy, and non-proliferative diabetic retinopathy.

	Control μL/min (*n* = 94)	DM no DR μL/min (*n* = 110)	NPDR μL/min (*n* = 56)	ANOVA *p*	Control vs DM no DR *p*	Control vs NPDR *p*	DM no DR vs NPDR *p*
<30 μm (*n* = 34)	0.05–0.31, 0.16 (23)	0.13–1.07, 0.42 (11)	n/a	0.019			
31–40 μm (*n* = 62)	0.25–1.04, 0.58 (20)	0.37–1.16, 0.70 (31)	0.12–0.71, 0.48 (11)	0.004	0.084	0.347	0.005
41–60 μm (*n* = 75)	0.62–2.95, 1.59 (15)	0.99–3.85, 2.13 (36)	0.32–2.59, 1.43 (24)	0.000	0.007	0.777	0.007
>61 μm (*n* = 89)	1.91–12.44, 6.50 (36)	3.05–11.12, 6.03 (32)	1.91–10.65, 4.86 (21)	0.061	0.719	0.049	0.224

Blood flow, expressed as range in μL/min, mean (*n*), comparing different vessel diameters across groups. *n* equals number of measured vessel segments. *p*-values were obtained using ANOVA and post-hoc tests. Abbreviations: DM = diabetes mellitus, DR = diabetic retinopathy, NPDR = non-proliferative diabetic retinopathy, n/a = not applicable.

**Table 6:** Summary of arteriolar blood flow by vessel diameter groups, comparing healthy controls, diabetes without retinopathy, and non-proliferative diabetic retinopathy.

	Control μL/min (*n* = 55)	DM no DR μL/min (*n* = 79)	NPDR μL/min (*n* = 47)	ANOVA *p*	Control vs DM no DR *p*	Control vs NPDR *p*	DM no DR vs NPDR *p*
<30 μm (*n* = 13)	0.06–0.29, 0.16 (9)	0.13–0.40, 0.30 (4)	n/a	0.032			
31–40 μm (*n* = 34)	0.40–0.85, 0.60 (9)	0.45–1.02, 0.73 (18)	0.12–0.71, 0.50 (7)	0.031	0.244	0.570	0.032
41–60 μm (*n* = 57)	0.85–2.95, 1.65 (9)	1.02–3.85, 2.33 (28)	0.48–2.59, 1.51 (20)	0.001	0.051	0.872	0.001
>60 μm (*n* = 77)	1.91–12.44, 6.68 (13)	3.2–11.12, 6.02 (14)	2.1–10.65, 4.96 (10)	0.068	0.578	0.054	0.314

Blood flow, expressed as range in μL/min, mean (*n*), comparing different arteriolar diameters across groups. *n* equals number of measured vessel segments. *P* values were obtained using ANOVA and post-hoc tests. Abbreviations: DM = diabetes mellitus, DR = diabetic retinopathy, NPDR = non-proliferative diabetic retinopathy, n/a = not applicable.

**Table 7:** Summary of venular blood flow by vessel diameter groups, comparing healthy controls, diabetes without retinopathy, and non-proliferative diabetic retinopathy.

	Control μL/min (*n* = 39)	DM no DR μL/min (*n* = 31)	NPDR μL/min (*n* = 9)	ANOVA *p*	Control vs DM no DR *p*	Control vs NPDR *p*	DM no DR vs NPDR *p*
<30 μm (*n* = 24)	0.05–0.33, 0.18 (16)	0.17–1.07, 0.47 (8)	n/a	0.043			
31–50 μm (*n* = 32)	7.13–13.58, 9.40 (12)	8.29–17.75, 11.51 (15)	3.21–9.05, 5.98 (5)	0.059	0.542	0.246	0.048
> 50 μm (*n* = 23)	1.86–11.62, 4.80 (11)	1.99–7.68, 3.85 (8)	1.10–2.87, 1.67 (4)	0.119	0.693	0.100	0.337

Blood flow, expressed as range in μL/min, mean (*n*), comparing different venular diameters across groups. *n* equals number of measured vessel segments. *p* values were obtained using ANOVA and post-hoc tests. Abbreviations: DM = diabetes mellitus, DR = diabetic retinopathy, NPDR = non-proliferative diabetic retinopathy, n/a = not applicable.

**Table 8:** Demographics and clinical measurements comparing low-flow diabetes without retinopathy and high-flow non-proliferative diabetic retinopathy eyes.

	DM no DR (*n* = 11)	NPDR (*n* = 6)	*p*
Parafoveal vessel density (%)			
SCP, mean ± SD	43.03 ± 4.98	43.70 ± 3.61	0.252
DCP, mean ± SD	50.71 ± 4.72	46.43 ± 3.17	0.417
Full retina, mean ± SD	53.81 ± 4.59	55.28 ± 1.95	0.123
OCTA SSI, mean ± SD	67.27 ± 3.77	69.83 ± 8.42	0.02
DM type			0.627
Type 1, *n* (%)	6 (55%)	3 (50%)	
Type 2, *n* (%)	5 (45%)	3 (50%)	
Disease duration in years, mean ± SD	10.87 ± 8.98	13.33 ± 8.02	0.624
HbA1c, mean ± SD	7.80 ± 2.34	6.77 ± 0.65	0.072
Age, mean ± SD	42.91 ± 16.22	40.17 ± 8.59	0.152

Abbreviations: DM = diabetes mellitus, DR = diabetic retinopathy, NPDR = non-proliferative diabetic retinopathy, SCP = superficial capillary plexus, DCP = deep capillary plexus, SD = standard deviation. *p*-value represents independent t-test or Chi-square test between DM without DR and NPDR.

**Table 9:** Summary of blood flow at a vessel bifurcation in healthy controls, diabetes without diabetic retinopathy, and non-proliferative diabetic retinopathy.

Flow (μL/min)	Healthy Control	DM without DR	NPDR
Daughter vessel 1	2.08	0.25	0.93
Daughter vessel 2	3.96	0.37	0.97
Parent vessel	6.03	0.61	1.98
